# Dysregulation of vitamin D synthesis pathway genes in colorectal cancer: A case‐control study

**DOI:** 10.1002/jcla.23617

**Published:** 2020-10-14

**Authors:** Hossein Sadeghi, Zeeba Kamaliyan, Roohollah Mohseni, Unes Sahebi, Ehsan Nazemalhosseini‐Mojarad, Naser Aghaei, Mohammad Reza Zali, Hamid Asadzadeh Aghdaei, Reza Mirfakhraie, Arfa Moshiri

**Affiliations:** ^1^ Molecular Genetics Department Genomic Research Center Shahid Beheshti University of Medical Sciences Tehran Iran; ^2^ Department of Medical Genetics School of Medicine Shahid Beheshti University of Medical Sciences Tehran Iran; ^3^ Clinical Biochemistry Research Center Basic Health Sciences Institute Shahrekord University of Medical Sciences Shahrekord Iran; ^4^ Student Research Committee Department of Clinical Biochemistry School of Medicine Shahid Beheshti University of Medical Sciences Tehran Iran; ^5^ Department of Gastrointestinal Cancer Gastroenterology and Liver Diseases Research Center Research Institute for Gastroenterology and Liver Diseases Shahid Beheshti University of Medical Sciences Tehran Iran; ^6^ Ophthalmology Department Ophthalmic Research Center Torfe Medical Center Shahid Beheshti University of Medical Sciences Tehran Iran

**Keywords:** colorectal cancer, *CYP27B1*, *CYP2R1*, single‐nucleotide polymorphism, vitamin D

## Abstract

**Background:**

The cytochromes P450 are a superfamily of enzymes that control the synthesis of the biologically active form of vitamin D, 1,25‐dihydroxyvitamin D3. These enzymes contribute to the formation of 1,25‐dihydroxyvitamin D3, which starts with a 25‐hydroxylation by *CYP2R1* and *CYP27A1* and a subsequent 1α‐hydroxylation via *CYP27B1*.

**Methods:**

By using quantitative real‐time polymerase chain reaction (qRT‐PCR), we analyzed the expression ratio of *CYP2R1*, *CYP27A1* and *CYP27B1* genes within the vitamin D metabolic pathway in a total of 75 colorectal cancer (CRC) tissues compared to the adjacent tissues. Furthermore, we evaluated the association of *CYP27B1* rs4646536 and *CYP2R1* rs12794714 and rs10766196 polymorphisms with CRC risk in a total of 490 subjects, including 245 CRC patients and 245 non‐cancer controls. The genotyping was performed using tetra‐primer amplification refractory mutation system polymerase chain reaction (TP‐ARMS–PCR) method.

**Results:**

The results indicated 2.3 and 2.7 upregulation of *CYP2R1* and *CYP27B1* genes in colorectal cancer tissues compared to the adjacent tissues, respectively. Rs12794714 AG genotype increased the risk of CRC (*P* = .03). Furthermore, a significant association was observed under the dominant inheritance model (*P* = .039).

**Conclusion:**

*CYP2R1* and *CYP27B1* genes were over‐expressed in CRC samples compared to the adjacent control tissues. Furthermore, *CYP2R1* rs12794714 variant was associated with the risk of CRC in the studied samples. *CYP2R1* rs10766196 and *CYP27B1* rs4646536 are not responsible for *CYP2R1* and *CYP27B1* genes expression alteration, respectively, but *CYP2R1* rs12794714 polymorphism may be the reason of *CYP2R1* upregulation and increased the risk of CRC.

## Graphical abstract

Patients and controls were recruited into this case‐control study, from Gastroenterology and Liver Diseases Research Center. Then, for the first step, total RNA was extracted from fresh frozen CRC tissues and the noncancerous tissues and the qRT‐PCR reaction was performed in 96‐well plates. The qRT‐PCR amplification efficiency was assessed using LinRegPCR software. In the second step, Genomic DNA was extracted from peripheral blood and SNPs frequencies were carried out by TP ARMS PCR. Analysis of inheritance models was performed using the online software.

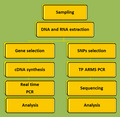

## 1 INTRODUCTION

According to the global cancer project estimation, colorectal cancer (CRC) is the second leading cause of cancer‐related death worldwide, accounting for around 1.8 million new cases and 860 000 deaths in 2018.^1^ The Iranian National Cancer Registry (IACR) Report shows that the incidence of CRC has increased during the last 25 years.^2^ Genetic factors, epigenetic alterations, and environmental factors may be associated with CRC progression.^3^, ^4^ Vitamin D is a steroid hormone and is involved in cell proliferation and differentiation.^5^ Multiple lines of evidence have shown the vitamin D pathway dysregulation in different types of cancer, for instance, cytochrome P450 (CYP) genes such as *CYP2R1*, *CYP27A1*, and *CYP27B1*.^6^, ^7^, ^8^ In summary, vitamin D synthesis is as follows: *CYP2R1* and *CYP27A1* convert vitamin D to circulating 25(OH)D. Then, *CYP27B1* converts 25(OH)D to 1, 25‐dihydroxyvitamin D, which is the biologically active form of the vitamin D.^9^ Increased concentrations of *CYP27A1* had been reported in the nuclei of normal colonic epithelia, aberrant crypt foci (ACF), and adenomatous polyps.^7^ The primary site of *CYP27B1* expression is in the proximal tubule of the kidney, but it is also expressed in other tissues such as the colon.^10^, ^11^ *CYP2R1* is expressed in colon; however, liver is the primary site of *CYP2R1* expression.^12^, ^13^ On the other hand, single‐nucleotide polymorphisms (SNPs) could modify the gene expression and, consequently, influence the risk of cancer.^14^ Additionally, numerous studies suggested that SNPs in the *CYP2R1* and *CYP27B1* were found to be associated with CRC risk.^15^, ^16^ Gong et al have shown the association between intronic *CYP27B1* rs4646536, located at the long arm of chromosome 12, and CRC risk. However, Dong et al did not find any significant association between this polymorphism and colon cancer.^15^, ^17^ Nam et al^18^ hypothesized that the *CYP2R1* rs12794714 and rs10766196 variants, both located at the CpG island, may modulate the gene transcription and were associated with the risk of developing type 1 diabetes. Furthermore, *CYP2R1* rs12794714 was found to significantly associate with CRC and serum level of 25(OH)D3.^16^ While several studies have investigated *CYP27B1* and *CYP2R1* expression and genetic association separately in CRC, only a few of them have simultaneously investigated the expression and genetic association of *CYP27B1* and *CYP2R1* with CRC. Hence, the current study aimed to investigate the relation between *CYP2R1*, *CYP27B1,* and *CYP27A1* expressions and CRC. We also intended to assess the contribution of *CYP2R1* and *CYP27B1* polymorphisms in the pathogenesis of CRC in a sample of Iranian population.

## 2 MATERIALS AND METHODS

### 2.1 Study population

Colorectal cancer patients were identified in endoscopy, oncology, or surgery clinics from July 2012 to December 2015. For DNA extraction, blood samples were obtained from 245 patients with sporadic colorectal cancer and 245 age‐ and sex‐matched cancer‐free controls and collected in ethylene‐diamine tetra acetic acid (EDTA) tubes. Patients with a history of hereditary or malignant diseases, and diseases affecting the digestive system were excluded from the study. For expression analysis, a total of 75 CRC fresh colorectal cancer tissues and adjacent control tissues were obtained from the Institute for Gastroenterology and Liver Diseases (Shahid Beheshti University of Medical Sciences). All specimens were immediately stored at −80°C until RNA extraction. Patients who had undergone radiotherapy or chemotherapy were excluded from expression analysis. This case‐control study was approved by the Gastroenterology and Liver Diseases Research Center Ethics Committee (Code: IR.SBMU.RCGLD.REC.1397.006). Written informed consent was obtained from all participants before entering the study.

### 2.2 RNA isolation and reverse transcription

Total RNA was extracted from fresh‐frozen CRC tissues and the noncancerous tissues using a GeneAll Hybrid‐R™ RNA purification kit (Geneall Biotechnology Co. Ltd) according to the manufacturer's protocol. The quantity and the quality of the total extracted RNA were estimated using NanoDrop^®^ ND‐1000 spectrophotometer (Thermo Fisher Scientific). The RNA purity was evaluated according to the A260/A280 ratio. The integrity of RNA was assessed by electrophoresis on a denaturing 1% agarose gel. Complementary DNA (cDNA) was reversely transcribed from 0.5 to 1 μg of DNase I‐treated RNA using a Revert Aid First‐Strand cDNA Synthesis Kit according to manufacturer instruction (Thermo Fisher Scientific).

### 2.3 Quantitative real‐time PCR

The quantitative real‐time PCR (qRT‐PCR) reaction was performed in 96‐well plates using 2.0X RealQ‐PCR Master Mix^®^ with SYBR Green (Ampliqon) on ABI StepOnePlus™ Real‐Time PCR Detection System (Applied Biosystems). All qRT‐PCR primers were designed by Allele ID 6 software (Premier Biosoft) and described in Table 1. Each reaction mixture consisted of 1 µL cDNA (10 ng), 10 µL 2X RealQ‐PCR Master Mix^®^, 1 µL (10 pmol/µL) of both forward and reverse primers, and 7 µL of PCR‐grade water, equating to a final volume of 20 µL. The beta‐2 microglobulin (*β2M*) mRNA was used as the reference gene. *β2M* was selected as the reference gene according to previous research for identification of housekeeping control genes in colorectal cancer.^19^ The thermal profile of the reaction was performed using the following conditions: initial denaturation at 95°C for 15 minutes; followed by 40 cycles at 95°C for 15 seconds and 60°C for 60 seconds followed by melting curve stage assessment. The melting curve profile and agarose gel electrophoresis were performed to verify the specificity of primers and the authenticity of the PCR products.

**TABLE 1:** List of primer pairs for qRT‐PCR

Genes	Forward primer	Reverse primer	Amplicon size (bp)
*CYP2R1*	CCGGGGCTGCCATTTATCG	AAACTGAAGATCTCTCCGTACACC	107
*CYP27B1*	GTCCAGACAGCACTCCACTC	ACCACAGGGTACAGTCTTAGC	137
*CYP27A1*	CACGACATCCAACACGCTGAC	CCACAGGGTAGAGACGCAGAG	185
*Beta‐2‐microglogulin*	TGTCTTTCAGCAAGGACTGGT	TGCTTACATGTCTCGATCCCAC	143

Abbreviation: bp, base pair.

### 2.4 DNA extraction and genotyping

Genomic DNA was extracted from peripheral blood leukocytes using the standard salting‐out procedure and stored at −20°C until further use.^20^ The primers used to amplify the target DNA sequences, which were designed by using Primer1 online software available from http://primer1.soton.ac.uk/primer1.html, are presented in Table 2. Three single‐nucleotide polymorphisms, including *CYP27B1* rs4646536 and *CYP2R1* rs12794714 and rs10766196, were amplified using polymerase chain reaction (PCR) contained 2 µL template DNA (100‐200 ng DNA), 12.5 µL Taq DNA Polymerase 2X Master Mix (Amplicon), 1 µL of each primer (10 pmol), and 6.5 µL DNase‐free water in a total volume of 25 µL. PCR cycling was performed on a GeneTouch thermocycler (BIOER). Amplification cycling reactions were carried out under the following conditions: an initial denaturation at 95°C for 5 minutes, followed by 32 cycles at 95°C for 30 seconds, annealing for 50 seconds at different temperatures (Table 2), and extension at 72°C for 1 minute, then a final incubation step at 72°C for 7 minutes. Finally, the products were separated via electrophoresis on 2% agarose gel containing RedSafe stain in 0.5× tris/borate/EDTA (TBE). To confirm the accuracy of genotyping, the randomly selected samples (10%) were re‐genotyped. SNPs had the following characteristics: (a) had a minor allele frequency greater than 10% and (b) were potentially functional predicted by in silico analysis.

**TABLE 2:** Primer sequences for *CYP27B1* rs4646536 and *CYP2R1* rs12794714 and rs10766196 genotyping

SNP	Primer	Primer Sequence (5′‐3′)	Amplicon size	Ta
*CYP27B1* rs4646536	FI primer	CTTCAGCCCCTAGCCTCATCTGGT	149 bp (C allele)	
	RI primer	TCTAGGTTGCAAAGCACAAAATGGAGAAAG	212 bp (T allele)	58°C
	FO primer	GCAACTAGTGGATGGAAGCAGGGAGAT	307 bp (Control)	
	RO primer	TAGGAGAGTGTTTGAGAACAGGGTTGGG		
*CYP2R1* rs12794714	FI primer	TTTCTCATGTAGACATGGGGAAGCGCA	152 bp (G allele)	
	RI primer	ATCTATTCCCTGGCAGCCTCAGCC	205 bp (A allele)	63°C
	FO primer	CATAAGTCCAACCAGGAAGGCCCTG	307 bp (Control)	
	RO primer	CGATGTGGAAGCTTTGGAGAGCTGAAGA		
*CYP2R1* rs10766196	FI primer	CTTGATTTTCCGACAAGCCGCGTTCG	127 bp (A allele)	
	RI primer	TCTGCCTCGCAGCTCTGTGGAATCT	187 bp (G allele)	63.6°C
	FO primer	AGCAAACGCCTACACCAGTCGTTCGTC	264 bp (Control)	
	RO primer	TCCGAGGCGATCCAGTCCTGATTTTCC		

Note

The nucleotide specificity is indicated in parentheses.

Abbreviations: bp, base pair; F, forward; I, inner; O, outer; R, reverse; SNP, single‐nucleotide polymorphism; Ta, annealing temperature.

### 2.5 Statistical analysis

Analysis of inheritance models and deviations from Hardy‐Weinberg equilibrium were performed using the online SNPStats program available from http://bioinfo.iconcologia.net/SNPstats and MEDCALC online software available from https://www.medcalc.org/calc/odds_ratio. The qRT‐PCR amplification efficiency was assessed using LinRegPCR software (version: 2017.1) and for each sample, the cycle threshold (Ct) and mean PCR efficiencies were determined. All the analyses were performed using SPSS 16.0 unless otherwise specified. The two‐tailed *t* test was used to analyze the association between the expression levels and the clinicopathological features of the CRC patients. *P* > .05 was considered statistically significant.

## 3 RESULTS

### 3.1 *CYP2R1*, *CYP27B1,* and *CYP27A1* expression levels in colorectal cancer tissues

**FIGURE 1:**
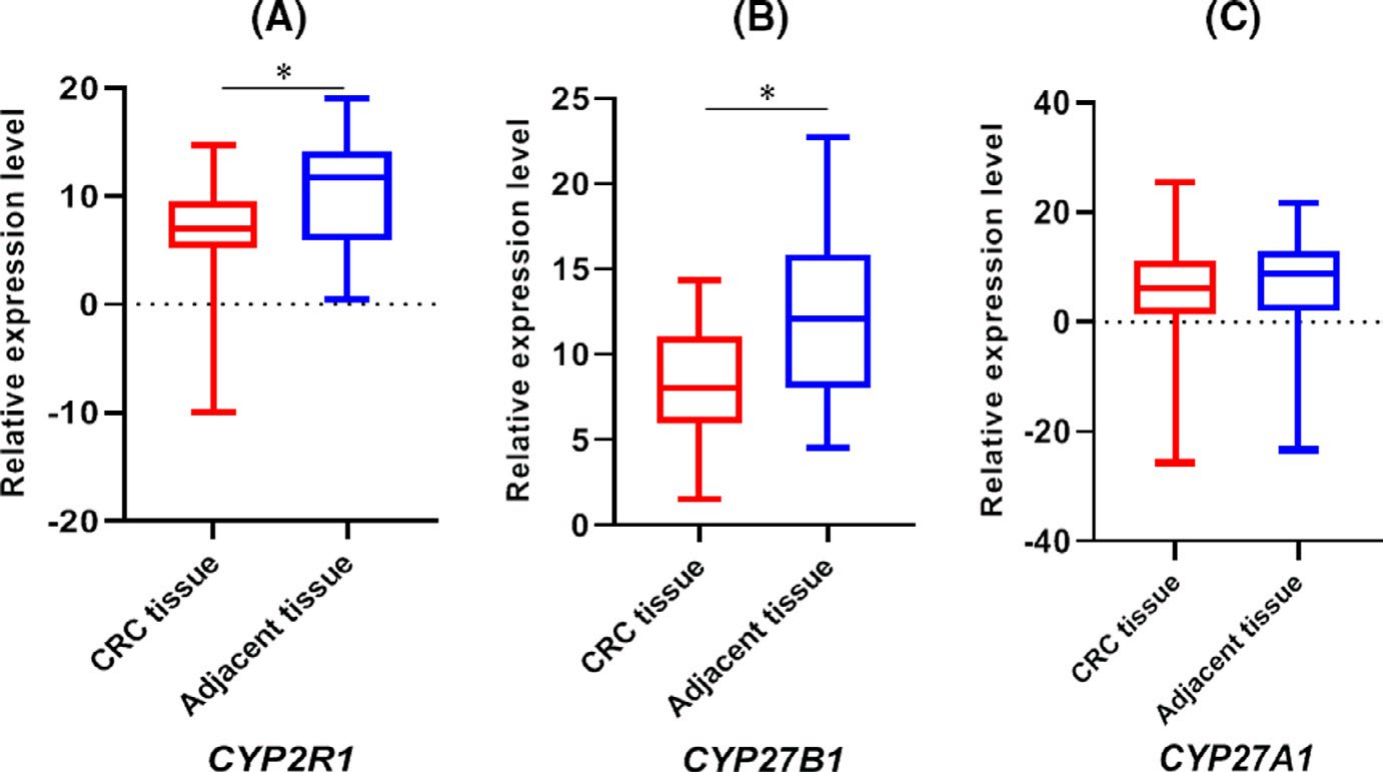
The relative expression levels (ΔCt) of *CYP2R1* (A), *CYP27B1* (B), and *CYP27A1* (C) genes in CRC samples compared to controls. A and B suggest that *CYP2R1* and *CYP27B1* genes were upregulated in CRC samples compared with controls. The mean ± standard deviation (SD) for *CYP2R1* CRC tissues and adjacent tissues were 8.31 ± 6.53 and 12.21 ± 5.85, respectively. The mean ± SD for *CYP27B1* CRC tissues and adjacent tissues were 7.78 ± 3.76 and 12.21 ± 5.59, respectively. As shown in C the expression of *CYP27A1* gene was not different between the studied groups. The mean ± SD for *CYP27A1* CRC tissues and adjacent tissues were 4.01 ± 14.73 and 5.07 ± 13.95, respectively. The relative expression levels comparison between CRC samples and controls was applied by paired *t* test. The asterisk sign (*) represents *P* < .05

We measured *CYP27B1*, *CYP27A1*, and *CYP2R1* expression levels using qRT‐PCR. Overall, the results indicated that 2.3 and 2.7 upregulation of *CYP2R1* and *CYP27B1* genes in colorectal cancer tissues compared to the adjacent tissues, respectively. *CYP2R1* and *CYP27B1* genes' expression was significantly upregulated in CRC tissues compared with adjacent tissues (Figure 1A,B). *CYP27A1* gene expression did not show any significant expression alteration when comparing the CRC tissues with adjacent tissues (Figure 1A). The correlations between the expression of *CYP2R1*, *CYP27B1*, and *CYP27A1* genes and clinicopathological features are shown in Table 3. None of the clinicopathological characteristics were significantly correlated with these genes.

**TABLE 3:** The correlation of *CYP2R1*, *CYP27B1,* and *CYP27A1* expression levels (ΔCt) and clinicopathological features of the CRC patients

Factors	No. of patients (%)	*CYP2R1*		*CYP27B1*		*CYP27B1*	
		Mean ± SD	*P* value	Mean ± SD	*P* value	Mean ± SD	*P* value
Age (y)							
>60	40 (53)	8.82 ± 3.6	.35	8.03 ± 4.12	.63	5.41 ± 5.15	.79
≤60	35 (47)	7.97 ± 4.01		7.87 ± 3.20		4.37 ± 4.24	
Gender							
Male	44 (59)	10.11 ± 3.54	.22	7.92 ± 3.81	.37	6.73 ± 6.22	.29
Female	31 (41)	8.93 ± 3.17		6.76 ± 3.43		4.06 ± 4.74	
Stage							
I & II	55 (73)	8.72 ± 8.45	.65	8.53 ± 4.33	.42	5.44 ± 9.38	.12
III & IV	20 (27)	9.77 ± 8.33		7.73 ± 5.84		2.75 ± 7.88	
Location							
Colon	50 (67)	7.17 ± 6.38	.48	8.64 ± 3.86	.12	4.93 ± 2.26	.19
Rectum	25 (33)	9.74 ± 5.83		6.40 ± 4.82		5.84 ± 3.08	

### 3.2 Genotyping

**FIGURE 2:**
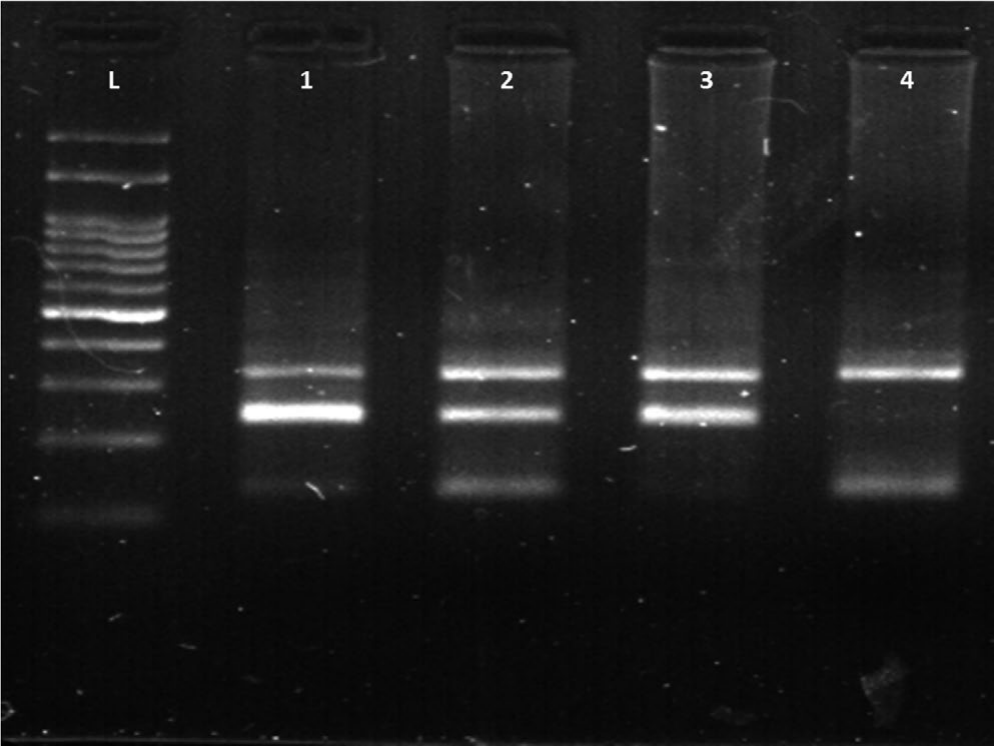
A representative 2% agarose gel electrophoresis of for identification of the *CYP2R1* rs12794714 genotypes. Lane L, 100 bp DNA ladder; lanes 1 and 2: AG genotype; lanes 3: AA genotype; and lanes 4: GG genotype

A total of 245 CRC patients (130 males and 115 females) with an average age of 54.87 ± 10.31 years and 245 cancer‐free controls (128 males and 117 females) with a mean age of 53.11 ± 11.22 years were analyzed in our study. All controls were from the same ethnic background (Iranian population). The CRC patients and normal controls were well matched concerning their ages and sex ratio (*P* > .05). Table 4 describes the demographic variables and clinical characteristics of the studied subjects. Our study investigated the association of *CYP27B1* rs4646536 and *CYP2R1* rs12794714 and rs10766196 polymorphisms with CRC risk in recessive and dominant inheritance models. Genotype and allelic frequencies of *CYP27B1* rs4646536 and *CYP2R1* rs12794714 and rs10766196 polymorphisms are shown in Table 5. The genotype distributions of *CYP27B1* rs4646536 and *CYP2R1* rs12794714 and rs10766196 polymorphisms were in Hardy‐Weinberg equilibrium (*P* > .05). Regarding the *CYP2R1* rs12794714, the AG genotype increased the risk of CRC (*P* = .03; odds ratio [OR] = 1.55; 95% confidence interval [CI]: 1.03‐2.34). In addition, the genotype frequencies showed significant differences under the dominant model (*P* = .03; OR = 1.50; 95% CI, 1.02‐2.21). There was no significant association between the *CYP27B1* rs4646536 and *CYP2R1* rs10766196 alleles and genotypes and CRC risk in any of the inheritance models (Table 5). Figure 2 represents 2% agarose gel electrophoresis for identification of CYP2R1 rs12794714 genotypes.

**TABLE 4:** Demographic data and clinical characteristics of the study participants for association study

Characteristics	CRC (n = 245) patients	non‐cancer controls (n = 245)
Median age, y	54.87 ± 10.31	53.11 ± 11.22
Gender		
Male	130	128
Female	115	117
Primary tumor location		
Colon	64%	
Rectum	25%	
Cecum	11%	
Differentiation		
Well‐differentiated	41%	
Moderately differentiated	22%	
Poorly differentiated	8%	
Not determined	29%	
Cigarette smoking		
No	74%	
Yes	26%	
Clinical stages, TNM		
I	16%	
II	48%	
III	30%	
IV	6%	

**TABLE 5:** Allele and genotype frequencies of *CYP27B1* rs4646536 *CYP2R1* rs12794714 and rs10766196 in CRC patients and controls. The SNPStats and MEDCALC online software were used for descriptive analysis and analysis of single SNPs: dominant and recessive inheritance models

Genotype/Allele	Case N (%)	Control N (%)	OR (95% CI)	*P* value
*CYP27B1* rs4646536 C>T				
T/T	117 (48)	111 (45)	1 (reference)	
C/T	96 (39)	94 (38)	1.03 (0.70‐1.52)	.87
C/C	32 (13)	40 (16)	1.32 (0.77‐2.24)	.31
T/T vs C/T+C/C			1.10 (0.77‐1.57)	.59
T/T+C/T vs C/C			1.30 (0.79‐2.15)	.31
T	316 (64)	330 (67)	0.88 (0.68‐1.15)	.35
C	174 (36)	160 (33)	1.14 (0.87‐1.48)	.35
*CYP2R* rs12794714 G>A				
G/G	64 (26.1)	85 (34.7)	1 (reference)	
A/G	131 (53.5)	112 (45.7)	1.55 (1.03‐2.34)	.03
A/A	50 (20.4)	48 (19.6)	1.38 (0.83‐2.31)	.21
G/G vs A/G‐A/A			1.50 (1.02‐2.21)	.039
G/G‐A/G vs A/A			1.05 (0.68‐1.64)	.82
G	259 (53)	282 (58)	0.83 (0.64‐1.06)	.14
A	231 (47)	208 (42)	1.21 (0.94‐1.56)	.14
*CYP2R* rs10766196 A>G				
A/A	97 (39.6)	90 (36.7)	1 (reference)	
A/G	127 (51.8)	127 (51.8)	0.93 (0.64‐1.35)	.39
G/G	21 (8.6)	28 (11.4)	0.70 (0.37‐1.31	.26
A/A vs A/G‐G/G			0.89 (0.62‐1.28)	.52
A/A‐A/G vs G/G			0.73 (0.40‐1.32)	.29
A	321 (66)	307 (63)	1.13 (0.87‐1.47)	.35
G	169 (34)	183 (37)	0.88 (0.68‐1.15)	.35

Note

*P *< .05 was considered statistically significant.

Abbreviations: CI, confidence interval; OR, odds ratio.

## 4 DISCUSSION

Vitamin D deficiency is inversely associated with the incidence and mortality of colorectal cancer.^21^ Under physiological conditions, *CYP27B1* and *CYP2R1* genes control the vitamin D synthesis, and vitamin D also controls the expression level of these genes.^9^, ^22^ *CYP2R1* and *CYP27B1* are dysregulated in different types of cancer, but the mechanism behind the dysregulation is not clearly defined.^23^, ^24^, ^25^, ^26^ In the current study, we first measured the expression levels of *CYP27A1*, *CYP27B1*, and *CYP2R1* in CRC samples. *CYP27B1* and *CYP2R1* expression ratios were significantly different when comparing CRC tissues with the adjacent tissues. In comparison with our results, dysregulated expression of *CYP2R1* and *CYP27B1* genes had been shown in different types of cancer, including oral squamous cell carcinomas and colorectal cancer.^6^, ^27^ We did not find any correlation between mentioned genes expression levels and clinicopathological characteristics in the studied patients. A number of inconsistent studies have shown that SNPs in both *CYP27B1* and *CYP2R1* genes may or may not be related to the CRC risk and circulating concentrations of vitamin D metabolites.^15^, ^16^, ^17^, ^28^, ^29^, ^30^ Furthermore, at the protein level, SNPs in the *CYP27B1* gene body could alter the *CYP27B1* enzymatic activity.^31^ To clarify whether variants in the vitamin D metabolism pathway were associated with CRC risk, we conducted a case‐control study to investigate the association between three SNPs in *CYP27B1* and *CYP2R1* genes and CRC risk. Regarding the *CYP2R1* rs12794714 polymorphism, AG genotype increased the risk of CRC in the patients (*P* = .03; odds ratio [OR] = 1.55; 95% confidence interval [CI], 1.03‐2.34). In addition, we observed that *CYP2R1* rs12794714 was associated with the risk of CRC under the dominant model (*P* = .039 OR = 1.50; 95% CI, 1.02‐2.21). In a previous study, Nam et al^18^ showed that the *CYP2R1* rs12794714 GG genotype and *CYP2R1* rs10766196 AA genotype were associated with the risk of developing type 1 diabetes in Korean children. Dorjgochoo et al^32^ also reported no association between *CYP2R1* rs12794714 polymorphism and the risk of breast cancer in Chinese women. In African Americans, *CYP2R1* rs12794714 was associated with a decreased risk of CRC.^16^ The AAGA haplotype of *CYP2R1* rs7936142‐rs12794714‐rs2060793‐rs16930609 was associated with a lower 25(OH)D concentration in the healthy Chinese population.^33^ Under the additive and recessive models, the exonic variant *CYP2R1* rs12794714 was significantly associated with plasma 25(OH)D concentration in northeastern Han Chinese children and people of Arab origin.^31^, ^34^ According to the HaploReg,^35^ RegulomeDB,^36^ and PROMO^37^ online databases, it is suggested that the *CYP2R1* rs12794714 variant may influence the binding affinity of several transcription factors, including ELF1, HNF4, SMARCA4, CDX2, and PEA3 in different cell types such as the Caco‐2 colon cancer cell line. In addition, according to rSNPBase,^38^ *CYP2R1* rs12794714 may affect the proximal and distal regulation and RNA binding protein‐mediated regulation. These transcription factors have important functions. HNF4α is a highly conserved transcription factor that plays a role as a tumor suppressor gene or an oncogene in colon cancer.^39^ CDX2 has a role in the progression of colorectal cancer and nominated as an independent prognostic factor in CRC. Moreover, it serves a clinically useful marker for epithelial neoplasms of the gastrointestinal tract. Previous researches showed that CDX2 might suppress colorectal tumorigenesis. Furthermore, CRC with advanced tumor stage and high tumor grade lost the CDX2 expression.^40^, ^41^ Pea3 is a prognostic marker in CRC, and the expression of Pea3 promotes the invasive and metastatic potential of colorectal carcinoma.^42^ Based on the results of our study, the distributions of *CYP27B1* rs4646536 and *CYP2R1* rs10766196 were not associated with the risk of CRC in any inheritance model (*P* > .05). Furthermore, these SNPs showed no significant differences in the allele and genotype frequencies when comparing CRC patients with healthy individuals (*P* > .05). In agreement with the results of our study, previous researches showed that there is no association between cases and controls in different types of cancer.^43^, ^44^ Conversely, in contrast to our study, in Chinese population, *CYP27B1* rs4646536 CC genotype reduced the risk of colorectal cancer compared with the TT genotype.^15^ Penna‐Martinez et al^45^ revealed that the rs10877012A/rs4646536T haplotype within the *CYP27B1* gene might be protective against papillary thyroid carcinoma. Dong et al^17^ suggested a lower risk of distal colon cancer among common allele homozygotes for *CYP27B1* rs4646536 T>C in the individuals with at least one copy of the *CYP24A1* rs2762942 C>T rare allele.

In conclusion, we showed that *CYP2R1* and *CYP27B1* genes were upregulated in CRC tissues compared to the adjacent control tissues. Besides, *CYP2R1* rs12794714 variant was associated with the risk of CRC in the studied samples. One of the limitations of this study was that the association and expression studies were performed on different samples, and we cannot correlate the tissue expression with the different genotypes. Furthermore, investigations with a larger sample size are recommended to estimate the role of rs12794714 variant in the expression of *CYP2R1*.

## AUTHOR CONTRIBUTIONS

Hossein Sadeghi performed experiments and analyzed the data. Zeeba Kamaliyan, Unes Sahebi, Roohollah Mohseni, and Ehsan Nazemalhosseini‐Mojarad performed experiments. Naser Aghaei, Mohammad Reza Zali, and Hamid Asadzadeh Aghdaei performed bioinformatic analyses and co‐wrote the article. Reza Mirfakhraie supervised the research, designed experiments, and co‐wrote the article. Arfa Moshiri supervised the research, designed experiments, and co‐wrote the article.

## Data Availability

The data that support the findings of this study are available from the corresponding author upon reasonable request.
